# Comparative transcriptomic analysis of transcription factors and hormones during flower bud differentiation in ‘Red Globe’ grape under red‒blue light

**DOI:** 10.1038/s41598-023-29402-5

**Published:** 2023-06-01

**Authors:** Xin Liu, Miao Yuan, Shizhuo Dang, Juan Zhou, Yahong Zhang

**Affiliations:** College of Agriculture, Ningxia University, Yinchuan, 750021 China

**Keywords:** Plant hormones, Transcriptomics

## Abstract

Grape is a globally significant fruit-bearing crop, and the grape flower bud differentiation essential to fruit production is closely related to light quality. To investigate the regulatory mechanism of grape flower bud differentiation under red‒blue light, the transcriptome and hormone content were determined at four stages of flower bud differentiation. The levels of indole-3-acetic acid (IAA) and abscisic acid (ABA) in grape flower buds at all stages of differentiation under red‒blue light were higher than those in the control. However, the levels of cytokinins (CKs) and gibberellic acid (giberellins, GAs) fluctuated continuously over the course of flower bud differentiation. Moreover, many differentially expressed genes were involved in auxin, CK, GA, and the ABA signal transduction pathways. There were significant differences in the *AUX/IAA*, *SAUR*, *A-RR*, and *ABF* gene expression levels between the red‒blue light treatment and the control buds, especially in regard to the *ABF* genes, the expression levels of which were completely different between the two groups. The expression of *GBF4* and *AI5L2* in the control was always low, while the expression under red‒blue light increased. *AI5L7* and *AI5L5* expression levels showed an upwards trend in the control plant buds and gradually decreased in red‒blue light treatment plant buds. Through weighted gene coexpression network analysis, we determined that the transcription factors *WRK48* (WRKY family), *EF110* (ERF family), *ABR1*, *CAMTA3* (CAMTA family), and *HSFA3* (HSF family) may be involved in the regulation of the *GBF4* gene. This study lays a foundation for further analysis of grape flower bud differentiation regulation under red‒blue light.

## Introduction

Grape (*Vitis vinifera* L.) is a globally significant fruit-bearing crop that is cultivated on all continents. Whether it is used for making fresh food or wine, it has major economic significance and is one of the most economically profitable fruit crops^1^. The quality and quantity of grape fruit are the most critical standards for grape cultivation and are directly affected by flower bud differentiation^2^. Flower bud differentiation of grape is an unusual process, compared with 1-year-old plants^3^. The shoot meristem generates both nutritional and reproductive structures, and the uncommitted primordia (also called anlagen) generated by the stem meristem differentiate into tendrils or inflorescences, which complete flowering in the second year^4^.

Grape flower bud differentiation is a very complex biological process that is influenced by the integration of the external environment and internal factors^5^. In the model plant *Arabidopsis thaliana*, flower development involves six pathways: the photoperiod, gibberellin, vernalization, autonomous, and ageing pathways, as well as the glycometabolism pathways, regulate floral-specific genes, resulting in the physiological transformation of the vegetative meristem into floral meristem^6^. These pathways converge to regulate the expression of flowering-related genes, such as *FLOWER LOCUS T* (*FT*), *CONSTANS* (*CO*), *SUPPRESSOR OF OVEREXPRESSION OF CO1* (*SOC1*), *LEAFY* (*LFY*), *APETALA1* (*AP1*), *APETALA2* (*AP2*), and *APETALA3* (*AP3*), and irreversibly induce the transformation from vegetative meristem to floral meristem^7^. Flower development pathway genes or genes involved in photoperiod or vernalization have also been identified in the grape genome; most of these genes are flowering signal integrators, floral meristem recognition genes, and floral organ recognition genes, such as MADS-box family genes and *VvFT/TFL1* family genes promoting early flowering^8^. The orthologue of *VvFT*, the *Arabidopsis thaliana* gene *FLOWERING LOCUST*, is related to seasonal flowering induction in latent buds, inflorescence and flower development^9^. The expression of *VvLFY* is related to inflorescence and flower developmen^10^. Homologues of *VvFUL* and *VvAP1* appear in the early stages of lateral meristem development and are maintained in both inflorescence and tendril primordia^11,12^.

As an essential environmental factor, light drives photosynthesis, participates in most processes regulating the plant life cycle, and plays a key role in plant flower bud differentiation^13^. Light quality is an important signal affecting plant morphogenesis and flower bud formation^14^. Plants perceive the quality of light through photoreceptors. Photoreceptors are classified as any of the following: photosensitive pigments (Phys) (for absorption of red/far-red light), cryptochromes (Crys) (for absorption of blue/ultraviolet A (UV-A) light), phototaxis proteins, Zeitlupe (ZTL/FKF1/LKP2) family members, and the UV-B absorbing UVR8 family members^15–17^. Photosensitizers and cryptochromes are responsible for plant morphological and developmental changes^18,19^. Therefore, research on the effect of light quality on plant growth and development mainly focuses on red and blue light. In addition, the combination of red and blue light forms a spectral absorption peak suitable for plant photosynthesis and morphogenesis, forming fully developed flower buds and increasing the number of buds^20,21^. Therefore, two wavelengths (blue and red) are necessary for plants.

Although some progress has been made in studying the grape flower bud differentiation process^22,23^, the regulatory mechanism of grape flower bud differentiation under red‒blue light is still unclear. In the early stages of this study, we screened red‒blue light for suitablity to grape flower bud differentiation, since it is necessary to study the flower bud differentiation process under red‒blue light if we wish to understand the regulatory mechanism of flower bud differentiation under this particular type of ligh. Therefore, in this study we used the screened red‒blue light for supplementary light treatment, with conditions of no supplementary light as the control, and the hormone content at different stages of flower bud differentiation was determined by high-performance liquid chromatography (HPLC). The differentially expressed genes (DEGs) significantly involved in flower bud differentiation were analysed by comparative transcriptomics. The transcription factors involved in the regulation of critical genes in flower bud differentiation were analysed by using weighted gene coexpression network analysis (WGCNA). To explore the regulation mechanism of grape flower bud differentiation under red‒blue light. This study provides preliminarily clarification of the regulatory mechanism for grape flower bud differentiation under red‒blue light and lays the foundation for further research.

## Results

### Flower bud differentiation of ‘Red Globe’ grape under red–blue light

**Figure 1:**
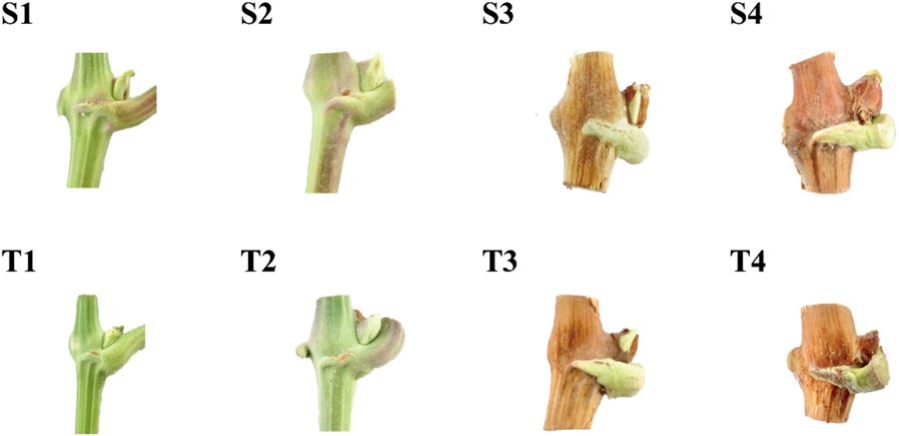
Flower buds of grapes at different developmental stages. (S1–S4) Four stages of flower bud differentiation under red–blue light. T. (T1–T4) Four stages of flower bud differentiation under control.

Before the transcriptome study, we conducted a morphological analysis of the floral bud development process (Fig. 1). In the first stage (April 30), the budding body was a flat green, the growth point was conical, the bud scale was not lignified, the stage one control (T1) branch diameter was less than the diameter of the stage one LED red‒blue treatment (S1) branches, and the two groups of flower buds were at the anlagen or uncommitted primordium stage (Supplementary Fig. S1). In the second stage (June 30), the buds expanded, became green, and increased in size. The stage two LED red‒blue treatment (S2) branches began to turn from green to brown. Some buds on the stage two control (T2) and S2 branches were at the stage of anlagen differentiation for forming inflorescence primordia. In the third stage (July 30), the buds became plump and brown. Seventy percent of the flower buds on the stage three control (T3) branches and 90% on the stage three LED red‒blue treatment (S3) branches were at the stage of anlagen differentiation for forming inflorescence primordia. The stage four (September 15) LED red‒blue treatment (S4) branches had hard buds that were dark brown, with semilignified scales, and both the stage four control (T4) and the S4 buds were all in the stage of anlagen differentiation for forming inflorescence primordia.

### Changes in hormone content during flower bud development

**Figure 2:**
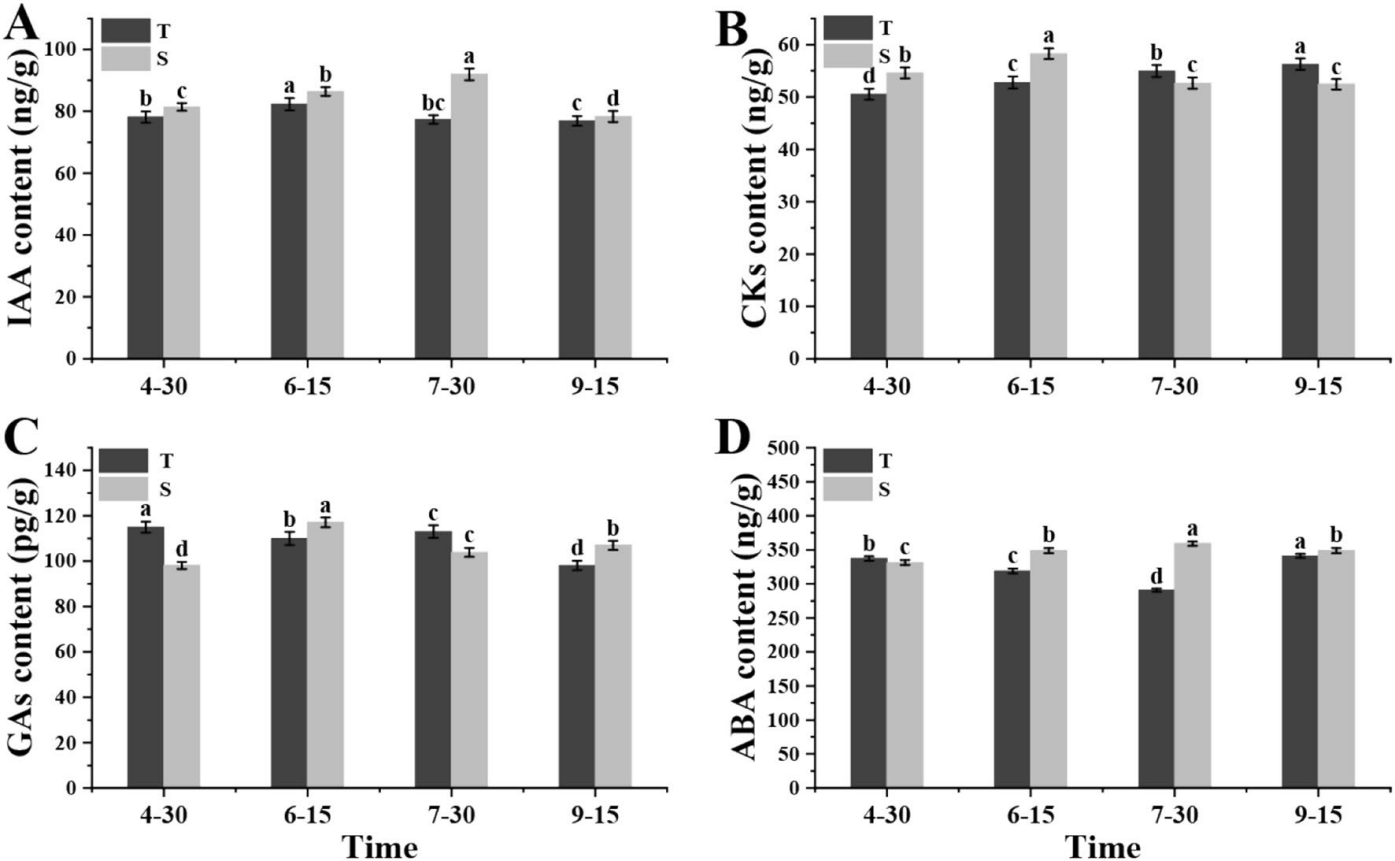
Changes of hormone content during flower bud development. (**A**) Content of IAA. (**B**) Content of CKs. (**C**) Content of GAs. (**D**) Content of ABA.

Flower bud differentiation is closely related to hormones. Therefore, the levels of indole-3-acetic acid (IAA), cytokinins (CKs), gibberellic acid (giberellins, GAs), and abscisic acid (ABA) in flower buds of the red‒blue light and control plants at the four stages were determined with HPLC. The HPLC analysis showed that the levels of these hormones changed significantly in different flower bud differentiation stages. The IAA content in the control was the highest in the second stage (Fig. 2A) and then decreased with development. Interestingly, the IAA content in the red‒blue light treatment was always higher than that in the control, reaching its highest level in the third stage and the lowest in the fourth stage. As shown in Fig. 2B, the trend of CK content in the two treatments was completely different. The CK content in the flower buds increased gradually under the control while under the red‒blue light treatment it was highest in the second stage and lowest in the fourth stage. GA content fluctuated throughout the flower bud differentiation stages. The levels in the control were higher than those in the red‒blue light treatment in the first and third stages of flower bud differentiation (Fig. 2C). The ABA content in flower bud differentiation was consistently lower in the control plants than in the red‒blue light treatment plants. In the third stage, the ABA content of the control plants was the lowest, and that of the red‒blue light treatment plants was the highest, with levels that were 24.6% higher than those of the control plants (Fig. 2D).

### Analysis of differentially expressed genes

**Figure 3:**
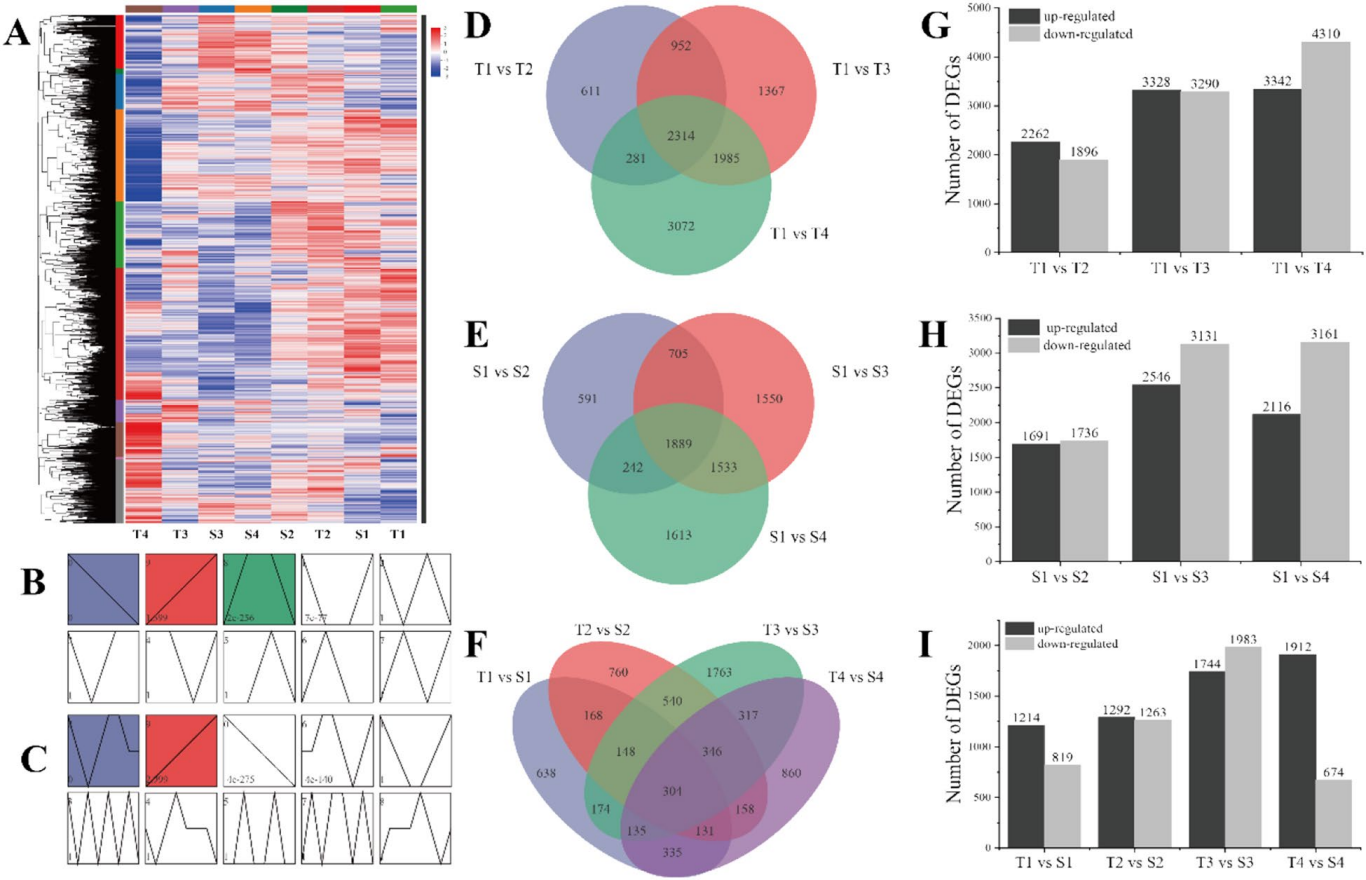
Expression analysis of DEGs in different developmental stages. (**A**) Clustering of DEGs. (**B**–**C**) Expression trends of DEGs in red-blue combination light treatment and control. (**D**) DEGs with red-blue combination light treatment development. (**E**) DEGs with control development. (**F**) DEGs between flower bud in different development stages. (**G**–**I**) Number of up- and down-DEGs.

We constructed a transcriptome sequencing library for the four stages of flower buds of the treatment and control groups, and 183.27 Gb clean reads were obtained. The clean reads of each sample were above 6.19 Gb, and the Q30 base percentage was above 93.28% (Supplementary Table S2). A principal component analysis (PCA) of the samples showed that the samples collected in this study had a high shared identity (Supplementary Figure S2). The DEGs were identified by using DESeq2 software, and the DEGs were found to have different expression trends in the red‒blue light treatment and the control plants (Fig. 3A–C). Compared with T1 grape buds, in T2, T3, and T4 buds 2314 common DEGs and 611, 1367, and 3072 specific expression genes were identified. There were 2262, 3328, and 3342 upregulated genes and 1896, 3290, and 4310 downregulated genes in T1 compared to T2 buds, T1 compared to T3 buds, and T1 compared to T4 buds, respectively (Fig. 3D,G). Compared with S1 buds, S2, S3, and S4 buds had 1889 common DEGs and 591, 1550, and 1613 unique expression genes. In S1 compared to S2 buds, S1 compared to S3 buds, and S1 compared to S4 buds, there were 1691, 2546, and 2116 upregulated genes and 1736, 3131, and 3161 downregulated genes, respectively (Fig. 3E,H). An analysis of T1 compared to S1 buds, T2 compared to S2 buds, T3 compared to S3 buds, and T4 compared to S4 buds showed that 1214, 1292, 1744, and 1912 genes were upregulated, and that 819, 1263, 1983, and 674 genes were downregulated at the four different maturation stages (Fig. 3F,I). In the third stage, the up- and downregulation of these genes increased significantly, indicating that these genes play a crucial regulatory role in the process of grape flower bud differentiation.

### Annotation of DEGs

We performed GO functional annotation and KEGG pathway analysis of the DEGs^24^. Through GO functional annotation, 6129 genes were annotated as 50 functional branches of cell components, biological processes, and molecular functions. Regarding all of the up- and downregulated DEGs, the first ten annotated GO terms were for binding (GO: 0,005,488), cellular component (GO: 0,044,464), catalytic activity (GO: 0,003,824), cellular process (GO: 0,009,987), metabolic process (GO: 0,008,152), membrane component (GO: 0,044,425), membrane (GO: 0,016,020), organelle (GO: 0,043,226), biological regulation (GO: 0,065,007) and response to stimulus (GO: 0,050,896). Among all the upregulated DEGs, 1995, 1902, 1853, 1673, 1668, 1394, 1173, 1102, 773, and 441 were annotated as these 10 GO terms. In the downregulated DEGs, the numbers were 1783, 1684, 1521, 1445, 1412, 1374, 1204, 953, 747 and 418, respectively (Supplementary Fig. S3, Supplementary Table S3).

**Figure 4:**
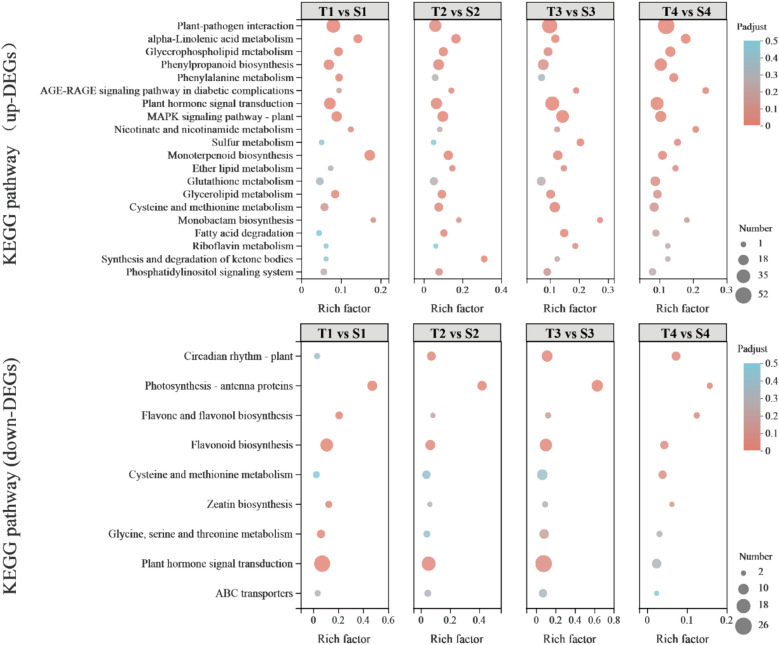
The pathways with the most significant Padjust. up-DEGs, Pathway enrichment analysis based on the differentially up-regulated genes between T and S. Down-DEGs, Pathway enrichment analysis based on the differentially down-regulated genes between T and S.

KEGG pathway analysis showed that the plant hormone signal transduction pathway (map04075) played an essential role in the grape flower bud differentiation. The plant hormone signal transduction pathway was enriched in the downregulated DEGs for the four stages under the red‒blue light treatment and the control. In addition, other enrichment pathways included those for flavonoid biosynthesis (map00941), flavonoid and flavanol biosynthesis (map00944), circadian rhythm-plant (map04712), and glycine, serine, and threonine metabolism (map00260). The plant hormone signal transduction pathway was also significantly enriched in the upregulated genes for the four stages under both the red‒blue light treatment and the control. In addition, the most enriched pathways were those for plant‒pathogen interactions (map04626), and other enrichment pathways included those for MAPK signalling pathway-plant (map04016), glycerophospholipid metabolism (map00564), phenylpropanoid biosynthesis (map00940), and monoterpenoid biosynthesis (map00902) (Fig. 4, Supplementary Table S4). The results showed that grape flower bud differentiation is a very complex biological process. In addition to the pathway for plant hormone signal transduction, the MAPK signal transduction, phenylpropanoid biosynthesis, flavonoid biosynthesis, and circadian rhythm-plant pathways are also involved in this process.

### DEGs related to plant hormone signal transduction

**Figure 5:**
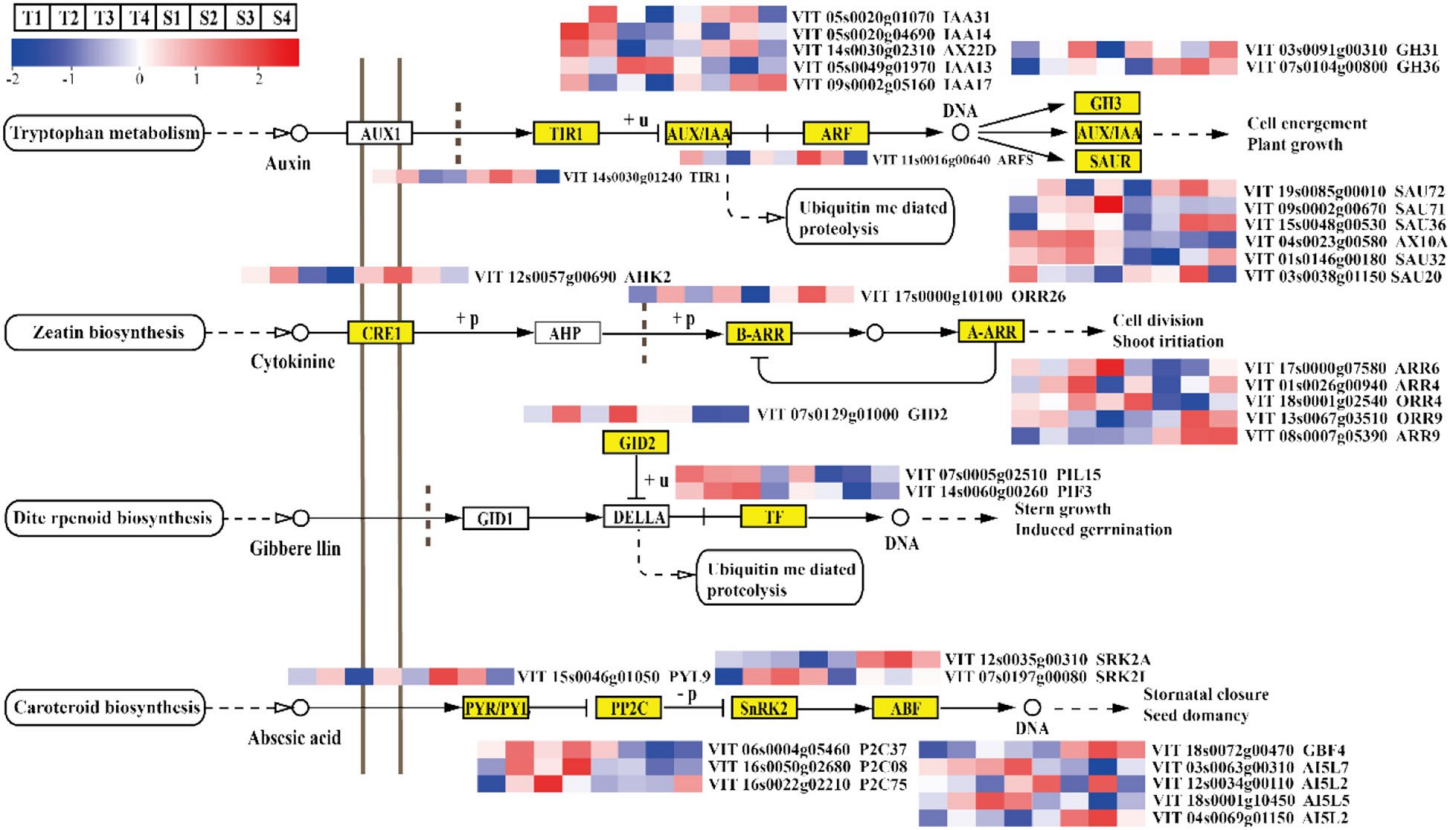
Expression of the plant hormone signal transduction pathway.

Previous studies have shown that grape flower bud differentiation is closely related to plant hormone signalling. In this study, we found that DEGs in plant hormone signal transduction are mainly concentrated in auxin, cytokinin, gibberellin, and abscisic acid pathways (Fig. 5). The 36 DEGs involved in these pathways include those involved in the auxin pathway, which are *TIR1* (VIT_14s0030g0124), *AUX/IAA* (VIT_05s0020g01070, VIT_09s0002g05160, VIT_05s0020g04690, VIT_05s0049g01970), *ARF* (VIT_11s0016g00640), *GH3* (VIT_07s0104g00800, VIT_03s0091g0031) and *SAUR* (VIT_19s0085g00010, VIT_09s0002g00670, VIT_15s0048g00530, VIT_01s0146g00180, VIT_03s0038g01150); cytokinin pathway *CRE1* (VIT_12s0057g00690), *B-ARR* (VIT_17s0000g10100), *A-ARR* (VIT_08s0007g05390, VIT_17s0000g07580, VIT_01s0026g00940, VIT_13s0067g03510, VIT_18s0001g02540); those involved in the gibberellin pathway, which are *GID2* (VIT_07s0129g01000), *TF* (VIT_07s0005g02510, VIT_14s0060g00260); and those involved in the abscisic acid pathway, which are *PYR/PYL* (VIT_15s0046g01050), *PP2C* (VIT_16s0022g02210, VIT_06s0004g05460, VIT_16s0050g02680), *SnRK2* (VIT_07s0197g00080, VIT_12s0035g00310) and *ABF* (VIT_18s0072g00470, VIT_03s0063g00310, VIT_18s0001g10450, VIT_04s0069g01150, VIT_12s0034g00110). Further analysis showed that the expression of the *TIR1*, *AUX/IAA*, and *ARF* genes was relatively high in the early stage of flower bud differentiation, while the expression was downregulated in the later stage of differentiation. The expression of *GH3* gradually increased during flower bud differentiation. In addition, the expression trend of the *SAUR* gene was completely different between the red‒blue light and the control plants. In the control plants, the expression of the *A-ARR* gene was high at the early stage of flower bud differentiation and then decreased, while the expression trend was opposite in the red‒blue light treatment plants. The expression trend of the *TF* gene was completely different in the control and red‒blue light treatment plants. In the control plants, *TF* gene expression levels were high at the early stage of flower bud differentiation and were low at the late stage, while in the red‒blue light treatment plants expression levels remained low throughout the flower bud differentiation stages. In the control plants, the expression of the *PP2C* gene increased over the course of flower bud differentiation, while in the red‒blue light treatment plants, the expression decreased. The expression trends of the *ABF* gene in the two groups were also different. The control plants’ expression of *GBF4* (VIT_18s0072g00470) and *AI5L2* (VIT_04s0069g01150) remained at a low level, while the expression in the red‒blue light treatment plants increased over the course of flower bud differentiation, reaching the highest expression levels in the third stage. Expression levels for *AI5L7* (VIT_03s0063g00310) and *AI5L5* (VIT_18s0001g10450) showed an upwards trend in the control plants but gradually decreased in the red‒blue light treatment plants. The results showed that these signalling genes played an important role in grape flower bud differentiation.

### qRT‒PCR validates the gene expression profiles

**Figure 6:**
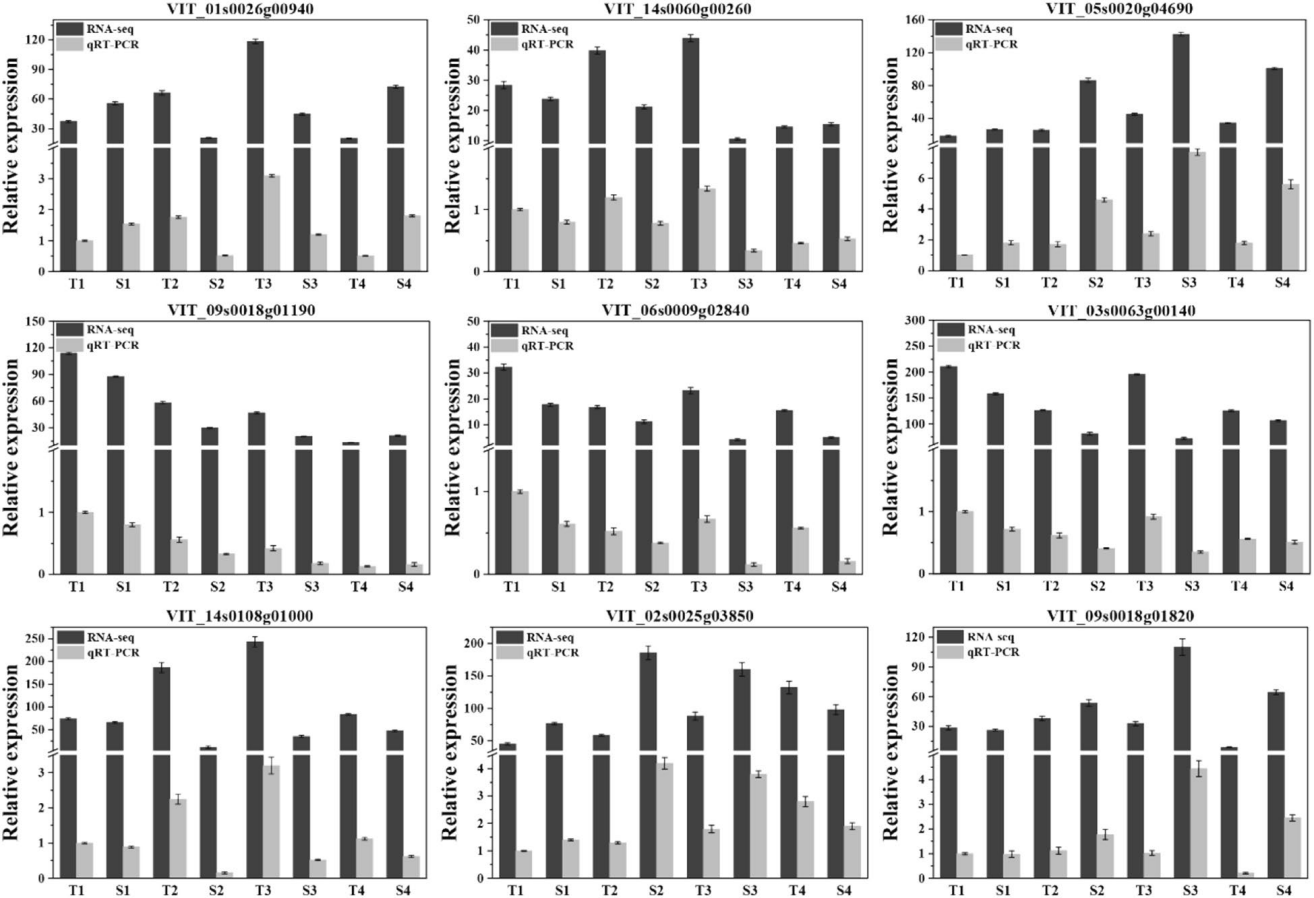
Validation of RNA-Seq data for 9 unigenes by qRT-PCR.

To validate our sequencing results, DEGs involved in plant hormone signal transduction, flavonoid biosynthesis and MAPK signal transduction pathways were randomly selected for qPCR analysis. The analysed DEGs included *ARR4* (VIT_01 s0026g00940), *PIF3* (VIT_14s0060g00260), *GBF4* (VIT_18s0072g00470), *HST* (VIT_09s0018g01190), *C75A1* (VIT_06s0009g02840), *CAMT* (VIT_03s0063g00140), *CML46* (VIT_14s0108g01000), *YODA* (VIT_02s0025g03850), and *M2K5* (VIT_09s0018g01820). The qRT‒PCR results showed that the expression patterns of these genes were consistent with those in the RNA-seq data, with a correlation coefficient of R = 0.82. These results showed that the gene expression pattern revealed by RNA-seq data was reliable and could be used for further analysis (Fig. 6).

### Screening through WGCNA for the transcription factors regulating grape flower bud differentiation

**Figure 7:**
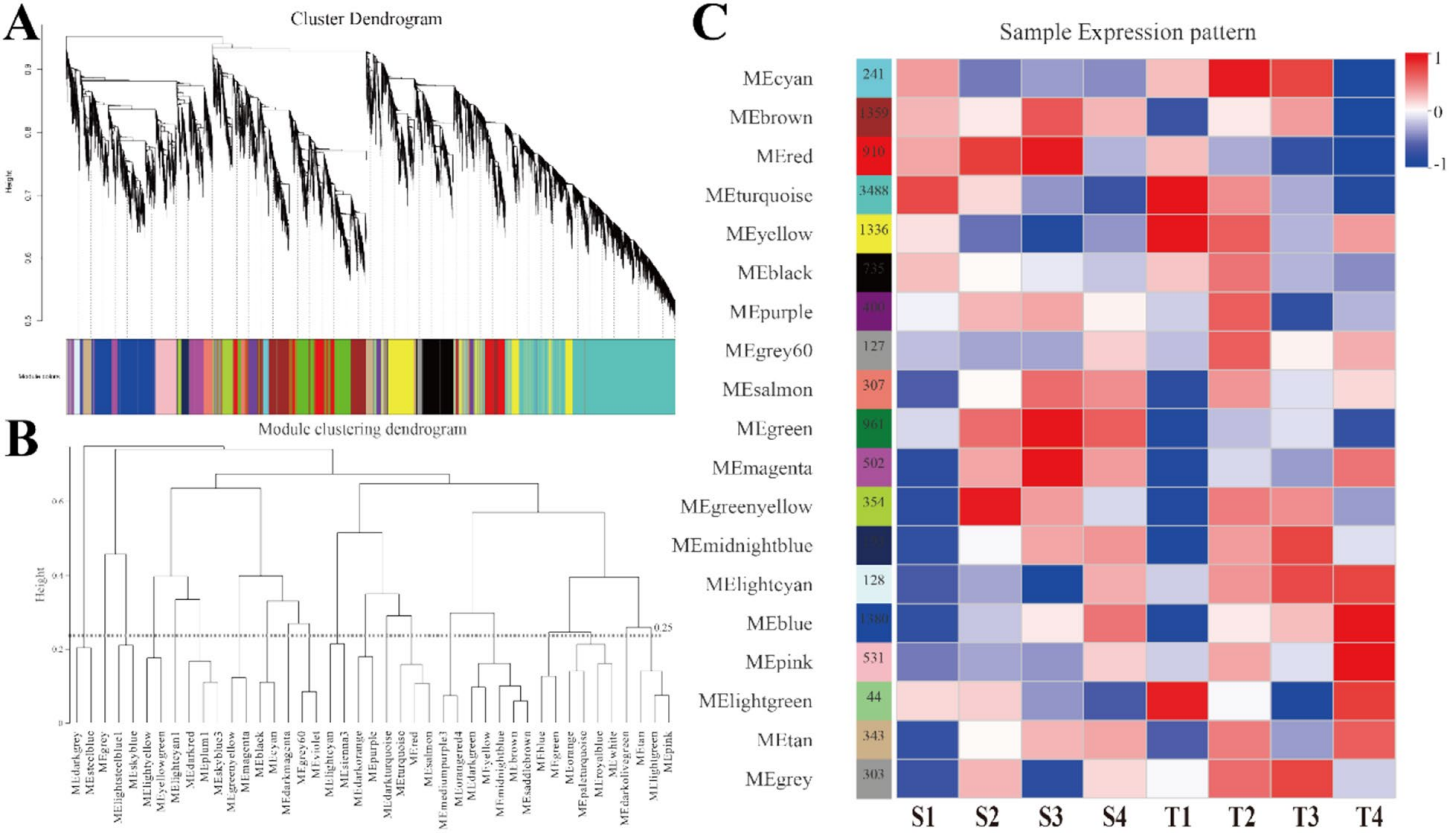
WGCNA of genes in grape flower bud differentiation. (**A**) Hierarchical cluster tree showing co-expression modules identified by WGCNA. (**B**) Module clustering dendrogram. (**C**) Module-tissue association.

In this study, a total of 24 samples from four stages of grape flower bud differentiation from the red‒blue light treatment and control groups were used for WGCNA (Supplementary Fig. S4). Through an assessment of the dynamic gene changes in different developmental stages and a correlation analysis between samples, the possible transcription factors regulating flower bud differentiation were investigated. First, the expression patterns of 25,844 DEGs extracted from transcriptome sequencing were analysed by using WGCNA, and the DEGs were divided into 19 modules (Fig. 7A, Supplementary Table S5) according to the similarity of their expression patterns. These modules could be divided into two main branches (one with two of the modules and the other 17 of the modules) (Fig. 7B). The correlation between the expression patterns of each module and different stages of grape flower bud differentiation was analysed. The results showed that the modules ‘red’, ‘green’ and ‘magenta’ were highly correlated with the third stage of the fastest flower bud differentiation under the red‒blue light treatment, indicating that these modules were closely related to the flower bud differentiation of grapes (Fig. 7C). A total of 2373 genes were found in these modules, and the gene association networks of 2373 genes were constructed. According to the degree of connection, the first 50 genes were considered hub genes. Among these 50 hub genes, seven transcription factors were found, including the ERF family *EF110* (VIT_18s0072g00260), *ABR1* (VIT_07s0031g01980), the bHLH family BH025 (VIT_00s0824g00020), *BH025* (VIT_00s0927g00010), the WRKY family *WRK48* (VIT_05s0077g00730), and *CAMTA3* (VIT_07s0141g00250), the NF-X1 family *NFXL2* (VIT_13s0067g00920) and the bZIP family *GBF4* (VIT_18s0072g00470) (Supplementary Fig. S5, Supplementary Table S6). As highly connected hub genes, these transcription factors may regulate flower bud differentiation in grape plants.

**Figure 8:**
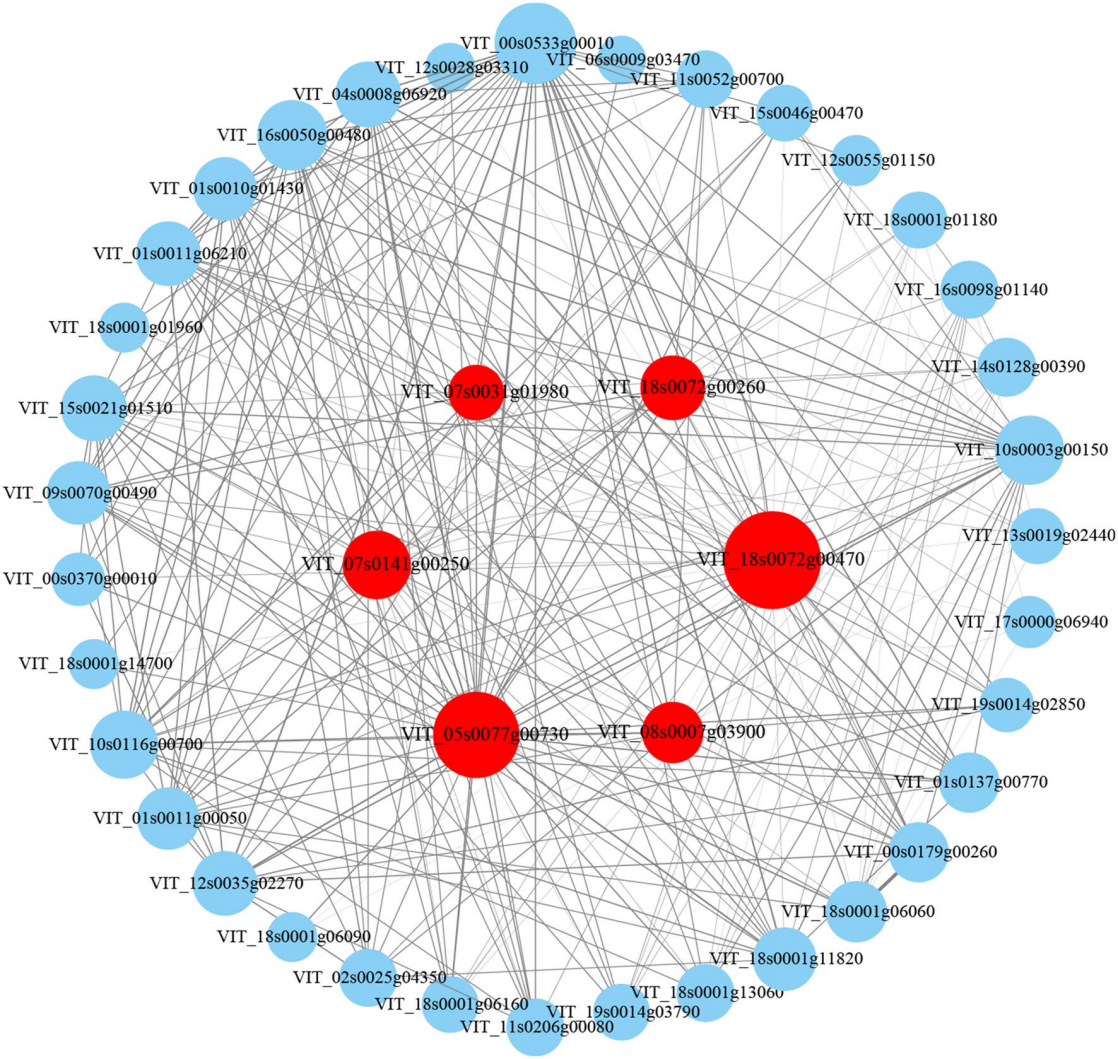
The correlation networks of genes in the ‘green’ module, in which only connected with *GBF4* gene (VIT_18s0072g00470) and edges weight ≥ 0.4 are displayed. Transcription factors and *GBF4* genes are shown in red, larger circles represent higher connectivity.

bZIP family genes are related to ABA signalling. To further analyse the transcription factors regulated by the GBF4 gene in the GA pathway, the genes related to *GBF4* in the ‘green’ module with edge weight (≥ 0.4) were selected for analysis (Fig. 8, Supplementary Table S7). It was found that 42 genes were regulated by *GBF4* (VIT_18s0072g00470), including the following hub transcription factors: the WRKY family *WRK48* (VIT_05s0077g00730), the ERF family *EF110* (VIT_18s0072g00260), *ABR1* (VIT_07s0031g01980), the CAMTA family *CAMTA3* (VIT_07s0141g00250), and the HSF family *HSFA3* (VIT_08s0007g0390), together with other non-hub transcription factor genes that have a certain regulatory relationship with the GBF4 gene.

## Disscussion

Grape yield depends on the amount of flower bud differentiation. Plant hormones are the key factors controlling the induction of flowering and interact with each other during plant developmen^25,26^. The types and levels of hormones in grape bud varied considerably at different developmental stages. In this study, the ‘Red Globe’ cultivar was used as the experimental material, and the supplemental treatment of red‒blue light was applied, with control conditions of no supplemental light. The four main plant hormones were identified in flower buds of both groups during four stages of flower bud differentiation. IAA was the first plant hormone discovered by researchers, and it is necessary for regular plant growth and development^27^. The IAA content in flower buds under red‒blue light treatment was higher than that in the control, and it showed a trend of increasing first and then decreasing over the course of flower bud differentiation, reaching its highest level at the third stage. Higher IAA content guarantees good flower bud formation, and this is consistent with the findings of previous studies^28^. CKs can promote the inflorescence development of lateral meristem in grape plants^29^. In our study, the content of CKs in flower buds increased during flower bud differentiation under the control, while the CK content decreased over the course of flower bud differentiation in the red‒blue light treatment plants. GAs are related to the initiation of flowering stage transitions and floral primordi^30,31^, which is a hot topic in current research^32^. Unlike CKs, GAs promote the differentiation of lateral meristem and inhibit the development of the inflorescence, which is beneficial to the development of the grape tendril. Grape plants with a mutation in GA-related genes are short in stature, with grape tendrils differentiated into inflorescences^33^. Interestingly, similar results were also observed in this study. The third stage of flower bud differentiation progressed the fastest, although this was less marked in the red‒blue light treatment plants than in the control. In addition, there was a significant difference in ABA content between normal and malformed flowers^34^. In this study, the ABA content in the red‒blue light treatment plants was higher than that in the control plants at the early stage of flower bud differentiation and was even 24.6% higher at the third stage, showing that ABA plays a vital role in flower bud differentiation. However, the regulation mechanism of hormone balance is complex. Like other perennial woody plants, grape flower bud differentiation requires a balance between hormones^35^.

In this study, 36 DEGs were annotated and identified as being involved in the auxin, cytokinin, gibberellin, and abscisic acid signal transduction pathways. In the auxin signal transduction pathway, the expression levels of *TIR1*, *AUX/IAA*, and *ARF* genes were relatively high at the early stage of flower bud differentiation, while they were downregulated at the late stage of differentiation, which was consistent with the findings of previous studies^36^. Studies have shown that *ARF*, combined with auxin response element *AUX/IAA*, plays a central role in transforming local auxin concentration into specific gene expression output, initiating flowering and regulating the development of floral organs^37,38^. In this study, four *AUX/IAA* genes and one *ARF* gene regulated the auxin pathway. The expression levels of the *IAA17* and *ARF*S genes in the red‒blue light plants was substantially inconsistent with expression levels in the control plants, indicating that these genes played an essential role in responding to flower bud differentiation under red‒blue light. In the cytokinin signal transduction pathway, the *ARR* gene participates in the circadian rhythm mechanism under light conditions, activates the function of flowering-induced gene *SOC1* to mediate the flowering of Arabidopsis^39^, and is highly expressed in branches with a high flowering rate in apple trees^40^. The expression levels of the six *ARR* genes identified in this study first increased and then decreased during flower bud differentiation and showed significant indigenous differences between the treatment and control groups, confirming the positive effect of *ARR* family genes on flower bud differentiation. *GID* and *TF* are genes that positively regulate the expression of *LFY, SOC1*, *FT*, *SPL*, and other genes that promote flower bud differentiation, which *DELLA* inhibits. The essential genes involved in flower bud differentiation in these gibberellin signal transduction pathways are also identified in this study. Unlike the results in previous studies, in this study the red–blue light treatment associated with a high flowering rate exhibited a lower expression of gibberellin than the control group, which may be due to the downregulation of gibberellin response genes without the effect of the *DELLA* gene^41^. The three signal components of *PYR/PYL*, *PP2C*, and *SnRK2* constitute the core ABA signalling pathway^42^, and positively regulate the flowering of long-day plants^43^. In addition, five *ABF* genes were also found to be involved in grape flower bud differentiation, indicating that *ABF* can be phosphorylated by *SnRK2*, affecting flower bud differentiation^44^.

WGCNA has been used to analyse data from numerous species to elucidate the interaction between physiological characteristics and gene expression^45,46^. In this study, WGCNA was used to evaluate transcript expression and trait changes at different stages of germination and differentiation under red–blue light, and three modules, ‘red’, ‘green’, and ‘magenta’ that were highly related to flower bud differentiation were screened. In these modules, eight transcription factors were identified as hub genes, including genes from the ERF family, bHLH family, WRKY family, CAMTA family, NF-X1 family, and bZIP family, suggesting that these transcription factors may be involved in grape flower bud differentiation under red‒blue light. Previous transcriptome sequencing studies found that transcription factors such as MYB, MADS-box, WRKY, ERF, and bHLH played an essential role in flower bud differentiation^47^. The MYB gene can regulate floral organ development and even mediate gibberellin signal transduction in flowering responses^48^, while the MADS-box gene is involved in the regulation of floral meristem initiation and development^49^. In addition, the bZIP and ERF genes are related to floral bud differentiation hormone signal response^50^. The bZIP family gene *GBF4* is a unique gene in the ABA signal transduction pathway. Thus far, there are few reports on this gene’s regulation of flower bud differentiation. We found that WRKY, ERF, CAMTA, and HSF family genes were involved in the transcriptional regulation of *GBF4* through WGCNA. WRKY family members were the key factors in the ABA response pathway, which could accelerate flowering by regulating *AP1*^51^, and HSF was involved in environmental stress response to regulate the flowering and development of plants^52^. The above transcription factors involved in plant hormone signal transduction regulation lacked clear evidence, but our study provided a theoretical basis for further confirming the regulatory mechanism of grape flower bud differentiation under red‒blue light.

## Conclusion

Overall, the comparative transcriptome analysis of the differential genes involved in the plant hormone signal transduction pathway showed significant differences in AUX/IAA, SAUR, A-RR, and ABF between red‒blue light treatment and control buds; this was especially true for the ABF genes, which were complete different in the two groups. The GBF4 (VIT_18s0072g00470) and AI5L2 (VIT_04s0069g01150) expression in the control remained low, while the expression under the red‒blue light treatment increased with the process of flower bud differentiation. AI5L7 (VIT_03s0063g00310) and AI5L5 (VIT_18s0001g10450) showed an upwards trend in the control and gradually decreased under red‒blue light. It is speculated that the difference in ABF family gene regulation plays a vital role in the grape flower bud differentiation response to red‒blue light. The WGCNA showed that the hub transcription factors WRK48 (VIT_05s0077g00730), ERF family EF110 (VIT_18s0072g00260), ABR1 (VIT_07s0031g01980), CAMTA family CAMTA3 (VIT_07s0141g00250), and HSF family HSFA3 (VIT_08s0007g0390) may be involved in the regulation of the GBF4 gene.

## Materials and methods

### Plant materials

The test material was 10-year-old *Vitis vinifera* cv. ‘Red Globe’ grape plants. The experiment was carried out in the growing season of 2021 in the sunlight greenhouse of Helan County Gardening Industrial Park, Yinchuan City. Our previous studies have shown that a ratio of red light:blue light = 4:1 (S) can significantly increase the flowering rate of ‘Red Globe’ grape plants (unpublished). Therefore, in this study, red light:blue light = 4:1 (S) LED plant lights were selected for the treatment group plants (the wavelengths of red and blue light were 620 and 435 nm) , and conditions of no supplementary light served as a control (T). Light tubes were placed at 30 cm above the test line plants, and the leaf light intensity was maintained at 300 μmol m^−2^ s^−1^ for 14 h per day from 8:00 to 22:00. In the first stage (T1 and S1, 30 April), the second stage (T2 and S2, 15 June), the third stage (T3 and S3, 30 July), and the fourth stage (T2 and S2, 15 September) of flower bud differentiation, 26 healthy branches were selected and removed with a sharp scalpel. Twenty buds were fixed in FAA (formaldehyde-acetic acid–ethanol) for paraffin sectioning, and the other buds were immediately frozen in liquid nitrogen and stored at -80℃ until further analysis.

### Physiological analysis of flower buds at different stages

Flower buds of four developmental stages were used for paraffin section making and hormone determination. Flower buds fixed in FAA were dehydrated in a gradient ethanol series and then were made transparent, wax-immersed, embedded, and patched in a dehydrator. Paraffin blocks containing flower buds were sliced with a semiautomatic slicer (Leica RM2245), stained with saffron-solid green, and sealed with neutral gum. Observation under an optical microscope (Olympus BX53) was performed using an SC180 image analysis system to obtain images^53^, and the flower bud differentiation stage was classified^54^. Auxins (IAAs), cytokinins (CKs), gibberellins (GAs), and abscisic acid (ABA) were quantified by using ultra-performance liquid chromatography spectrometry (ACQUITY H-Class, QDA), the mobile phase was methanol–water (containing 0.2% glacial acetic acid) with a volume ratio of 45:55. The flow rate was 0.25 mL min^−1^, the column temperature was 30 °C, and the injection volume was 10 μL^55^, with three biological replicates.

### RNA sequencing and expression analysis

The RNA of the samples was extracted using the OminiPlant RNA Kit (DNase I) (Beijing ComWin Biotech Co., Ltd. Beijing, China). Nanodrop2000 was used to detect the concentration and purity of RNA, agarose gel electrophoresis was used to detect RNA integrity, and Agilent2100 was used to determine the RNA integrity number (RIN) value for further quantitative real-time reverse transcription-PCR (qRT‒PCR) and RNA-seq determination. The Illumina HiSeq 2500 sequencing platform was used to perform RNA-seq. Raw reads were sequenced and then were processed to obtain clean reads of high quality. The clean reads after quality control were aligned with the reference genome (http://plants.ensembl.org/Vitis_vinifera/Info/Index) using HISAT2 software (Version 2.1.0)^56^ to obtain the mapped reads for subsequent analysis.

### GO and KEGG pathway enrichment analysis

Differential expression analysis of samples was carried out using the expression difference analysis software DESeq2 (Version 1.10.1)^57^. Differentially expressed genes between different samples were screened out using FPKM (Fragments Per Kilobases per Million reads) values and |log_2_fold changes|≥ 1, and the corrected Padjust < 0.05 as the screening standard to study the function of differential genes. We used the fastcluster (Version 1.2.4) package in R was used for cluster analysis of differentially expressed genes, hierarchical cluster analysis, and complete Gene clustering method. The DEGs in gene concentration were compared with data in the Gene Ontology (GO) and Kyoto Encyclopedia of Genes and Genomes (KEGG) databases to obtain gene functional annotation and related metabolic pathway information in different samples^58^.

### RNA-seq data confirmation through qRT-PCR

In this study, qRT‒PCR was used to validate the RNA-Seq results. Real-time fluorescence quantitative PCR was used to verify candidate DEGs related to hormone signal transduction and other metabolic pathways. The reference gene was *Actin1* (GenBank: XP_008654957.1). The primers (Supplementary Table S1) were designed by using Primer 6.0 software and were synthesized by Sangon (Shanghai, China). RNA and transcriptome sequencing samples for the same batch were conducted with qRT‒PCR using ChamQ Universal SYBR qPCR Master Mix kit (Vazyme Biotech Co., Ltd. Nanjing, China). The reaction procedure was as follows: reverse transcription at 50 °C for 5 min, predenaturation at 95 °C for 10 min, denaturation at 95 °C for 10 s, and annealing at 60 °C for 30 s, 40 cycles, and three biological repeats. The gene expression level was calculated by the 2^-ΔΔCt^ method.

### Gene network construction and visualization

To further study the hormone regulation mechanism and possible transcription factors in grape flower buds, we used the WGCNA (Version 1.63) package in R to construct the coexpression network^59^. The soft threshold of coexpression network clustering was selected according to the FPKM value of all genes in the sample; R^2^ > 0.8 was the norm, and the soft threshold was 9. All FPKM values were transformed into a topological overlap matrix (TOM), and each gene was clustered by hierarchical clustering^60^. The dynamic tree cutting method was used to divide genes into different coexpression modules. The minimum number of genes in each coexpression module was set at 30, and the coexpression modules with similar clustering were merged with 0.3 as the boundary. The correlation between different modules and the degree of genes in the module (module membership, MM) were calculated. Key modules related to flower bud differentiation were identified for subsequent analysis. Cytoscape (Version 3.8.0) was used to visualize the regulatory network mapping.

### Ethical standards

The conducted experiments comply with the laws of China.

## Supplementary Information

Supplementary Figures.

Supplementary Tables.

## Data Availability

Data supporting the results and conclusions are included in both the article and additional files. All the transcriptome data have been deposited in the NCBI Sequence Read Archive (SRA) under accession number: PRJNA843849 (http://www.ncbi.nlm.nih.gov/sra).
