# Supplementary material for: Comparative transcriptomic analysis of transcription factors and hormones during flower bud differentiation in ‘Red Globe’ grape under red‒blue light
Source: Sci Rep. 2023 Jun 1;13:8932. doi: 10.1038/s41598-023-29402-5 (PMC10235105; doi:10.1038/s41598-023-29402-5)
Supplement: Supplementary file 1 — Supplementary Figures. [file 41598_2023_29402_MOESM1_ESM.docx]

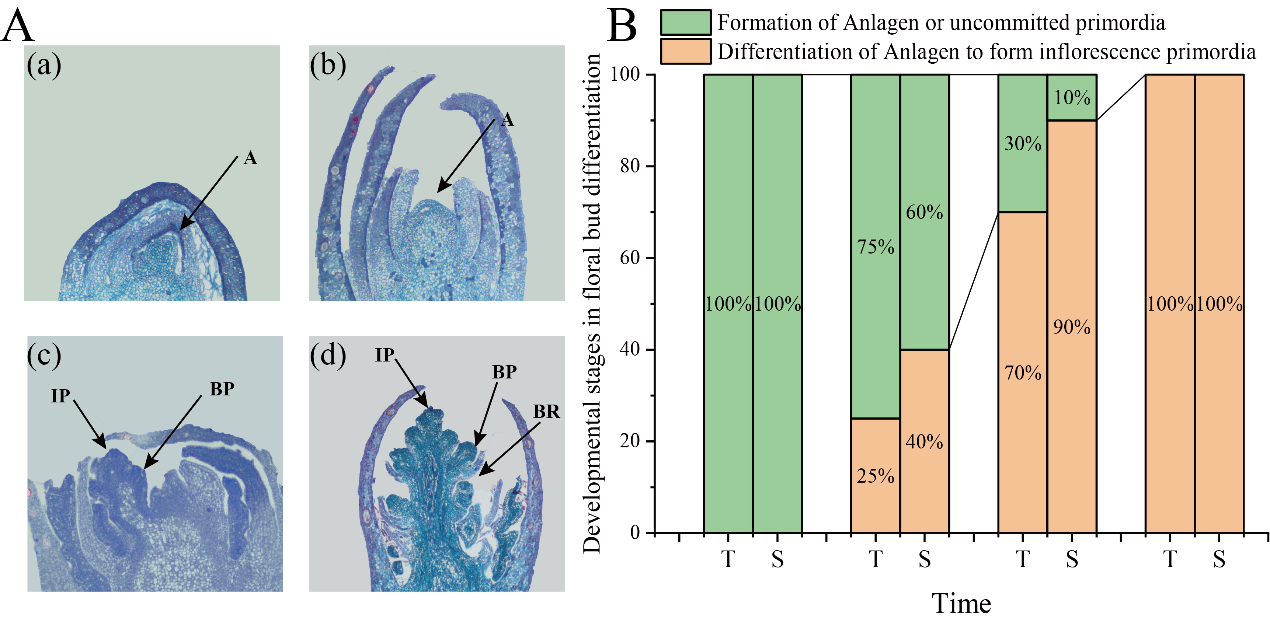


**Figure S1.** Development stages in flower bud differention. (**A**) Paraffin sections of flower buds, (**a**) and（**b**）are satge of formationof anlagen or uncommited primordia, (**c**) and (**d**) are satge of Differentiation of anlagen to form inflorescence primordia. (**B**) Stacked chart of development stages in floral bud differention
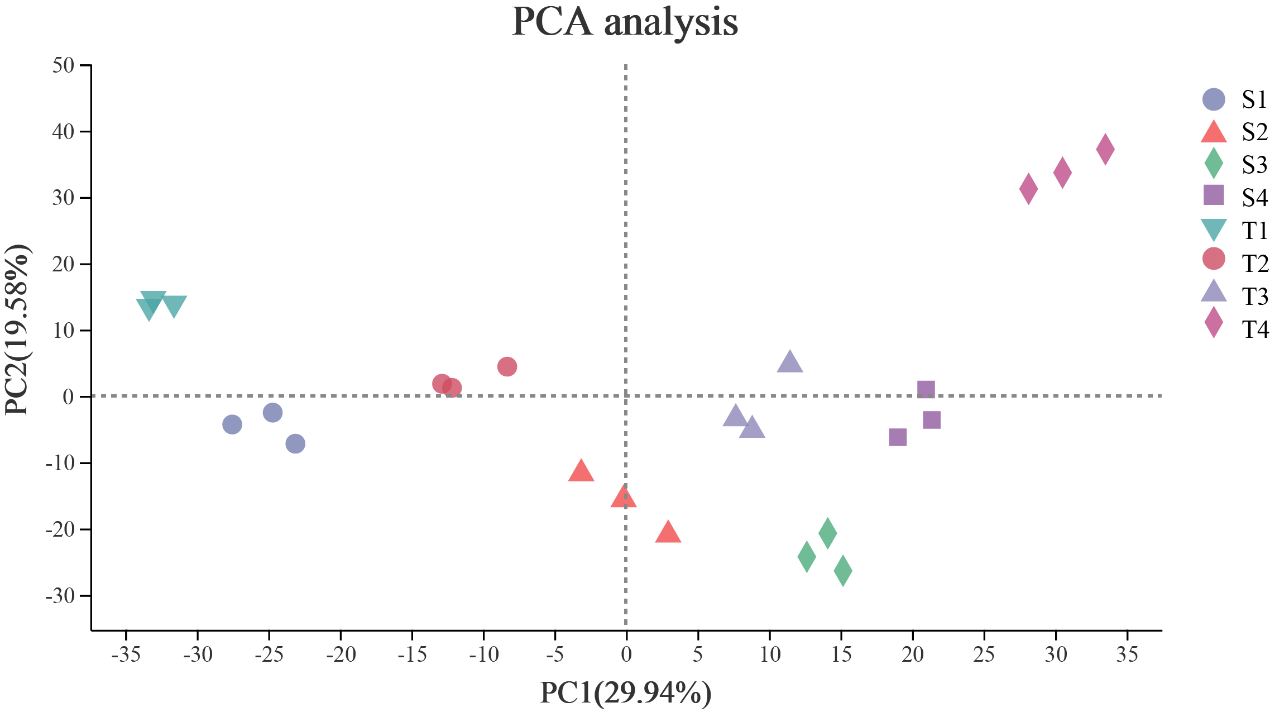


**Figure S2.** Principal component analysis of the different sample based on the gene expression profiles.


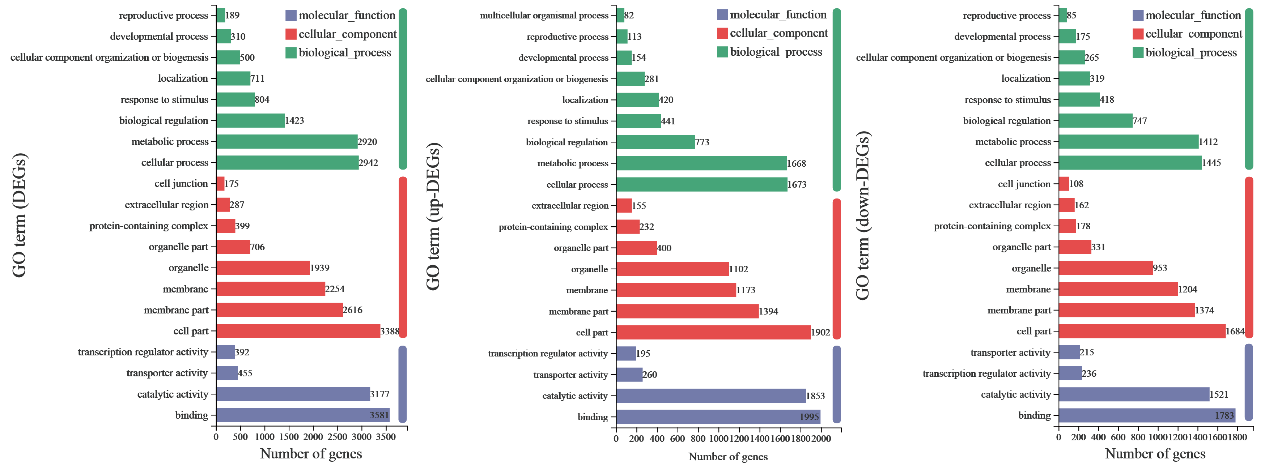


**Figure S3.** GO classification of DEGs. From left to right are total DEGs, up-regulated DEGs, down-regulated DEGs.


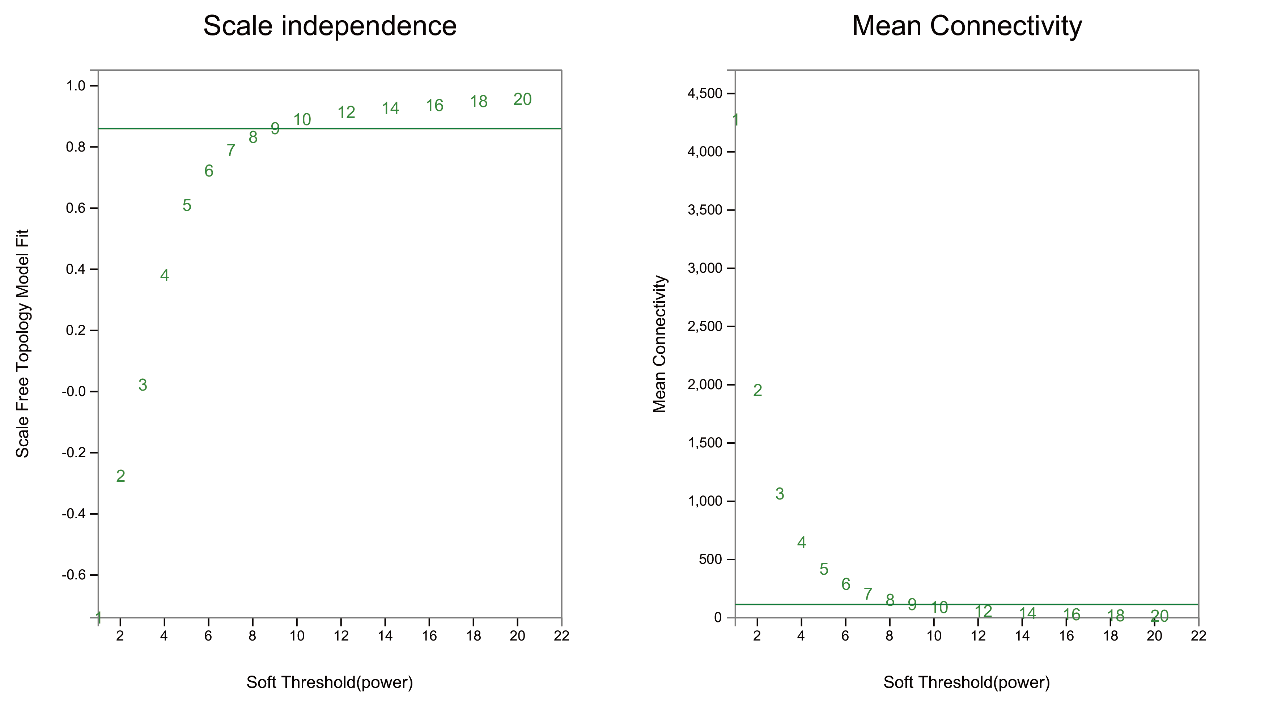


**Figure S4.** Scale independence and mean connectivity by calculating 25844 genes. Correlation relation was set 0.8 and β was 9.

**
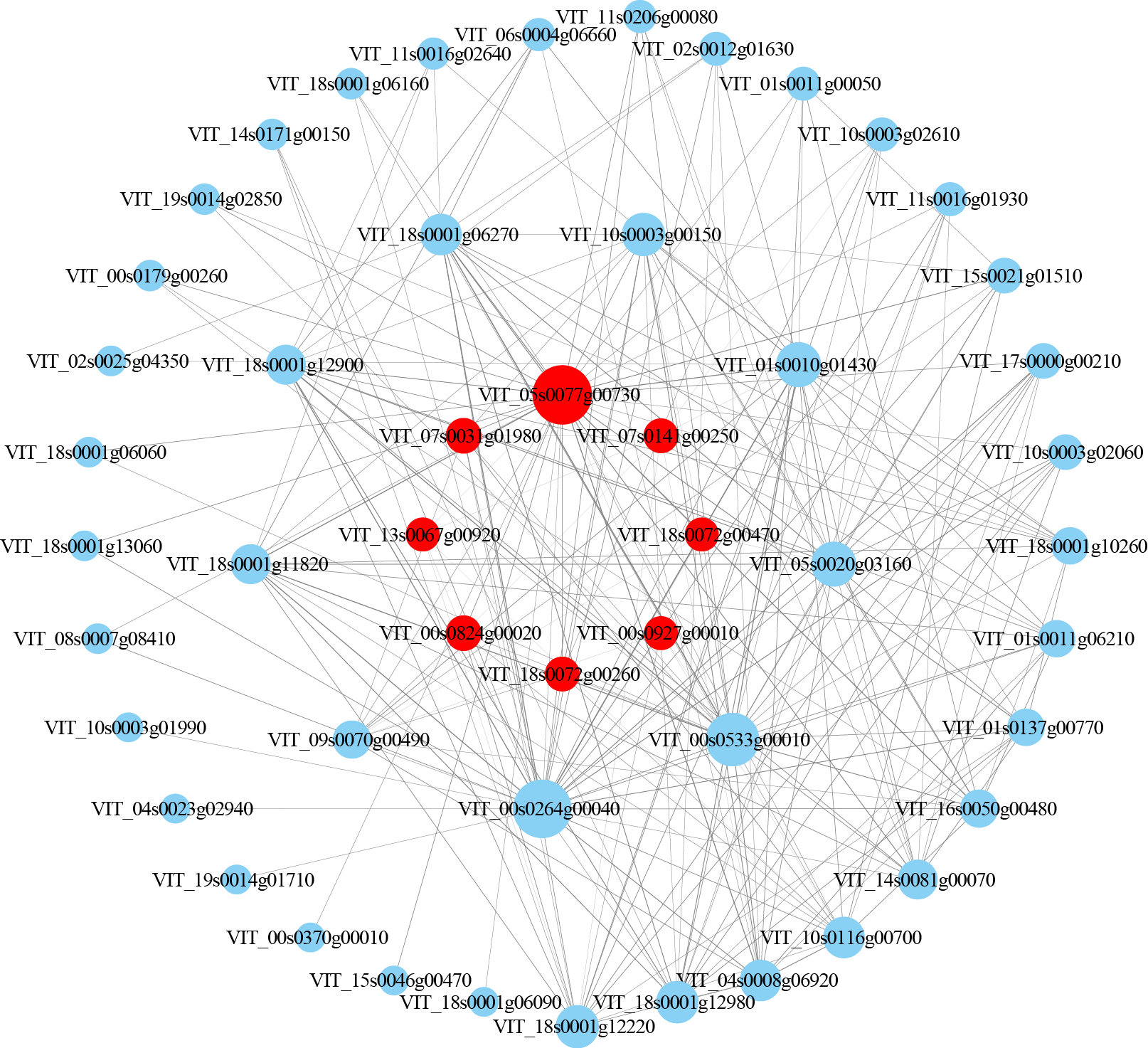
**

**Figure S5.** Co-expression network analysis of flower bud differentiation stage-specific modules.
